# High resolution microscopy reveals significant impacts of ocean acidification and warming on larval shell development in *Laternula elliptica*

**DOI:** 10.1371/journal.pone.0175706

**Published:** 2017-04-19

**Authors:** Christine H. Bylenga, Vonda J. Cummings, Ken G. Ryan

**Affiliations:** 1 School of Biological Sciences, Victoria University of Wellington, Wellington, New Zealand; 2 National Institute of Water and Atmospheric Research (NIWA), Wellington, New Zealand; Xiamen University, CHINA

## Abstract

Environmental stressors impact marine larval growth rates, quality and sizes. Larvae of the Antarctic bivalve, *Laternula elliptica*, were raised to the D-larvae stage under temperature and pH conditions representing ambient and end of century projections (-1.6°C to +0.4°C and pH 7.98 to 7.65). Previous observations using light microscopy suggested pH had no influence on larval abnormalities in this species. Detailed analysis of the shell using SEM showed that reduced pH is in fact a major stressor during development for this species, producing D-larvae with abnormal shapes, deformed shell edges and irregular hinges, cracked shell surfaces and even uncalcified larvae. Additionally, reduced pH increased pitting and cracking on shell surfaces. Thus, apparently normal larvae may be compromised at the ultrastructural level and these larvae would be in poor condition at settlement, reducing juvenile recruitment and overall survival. Elevated temperatures increased prodissoconch II sizes. However, the overall impacts on larval shell quality and integrity with concurrent ocean acidification would likely overshadow any beneficial results from warmer temperatures, limiting populations of this prevalent Antarctic species.

## Introduction

Human activities have resulted in a substantial increase in greenhouse gas emissions with atmospheric CO_2_ concentrations increasing by 40% to over 400 ppmv since the industrial revolution, and these are projected to reach 450 ppmv by 2100 [1,2]. Increased greenhouse gases raise surface temperatures, and subsequently lead to warmer oceans due to the uptake of atmospheric energy [1]. Increased partial pressure of CO_2_ (*p*CO_2_) can further impact marine environments through ocean acidification (OA) [3]. Oceanic pH has declined by 0.1 units since measurements began, with further reductions of over 0.3 units projected for the end of the century [1].

Ocean acidification may directly impact calcifiers due to reduced saturation states (Ωs) of calcium carbonate in seawater [4]. The Ω is dependent on calcium and carbonate concentrations, and their relative solubilities (*K’*_sp_) [4]. When undersaturation is reached (Ω < 1), dissolution of calcium carbonate occurs. Ω varies due to differing *K’*_sp_ among polymorphs of calcium carbonate [4]. Impacts of OA are particularly pressing in polar regions due to increased calcium carbonate solubility in cold water [4]. In these regions, the relatively soluble polymorph aragonite become undersaturated by 2050 [5], and even earlier in winter months in the Southern Ocean [6].

Increased pCO_2_ may also have metabolic impacts due to cellular acidosis or hypercapnia [7,8]. Invertebrates can respond to reduced pH by actively regulating their internal acid-base conditions, which they do by metabolic consumption or active transport of protons. However, these are likely to be short term solutions [7], as there would be an increased energetic cost to this process [9]. Bivalves are particularly sensitive to OA due to their low capacity to regulate the acid-base balance of their haemolymph, especially at vulnerable early stages [10]. Alternatively, internal acidosis can be buffered by bicarbonate, which is supplied through the dissolution of skeletal structures [7,11], resulting in reduced shell growth rates and integrity. Elevated temperatures can also affect rates of metabolism and development [12,13], survival and larval sizes [14,15]. Exposure to other stressors (including warming) may exacerbate or mitigate the effects of OA [8,14,16].

**Fig 1:**
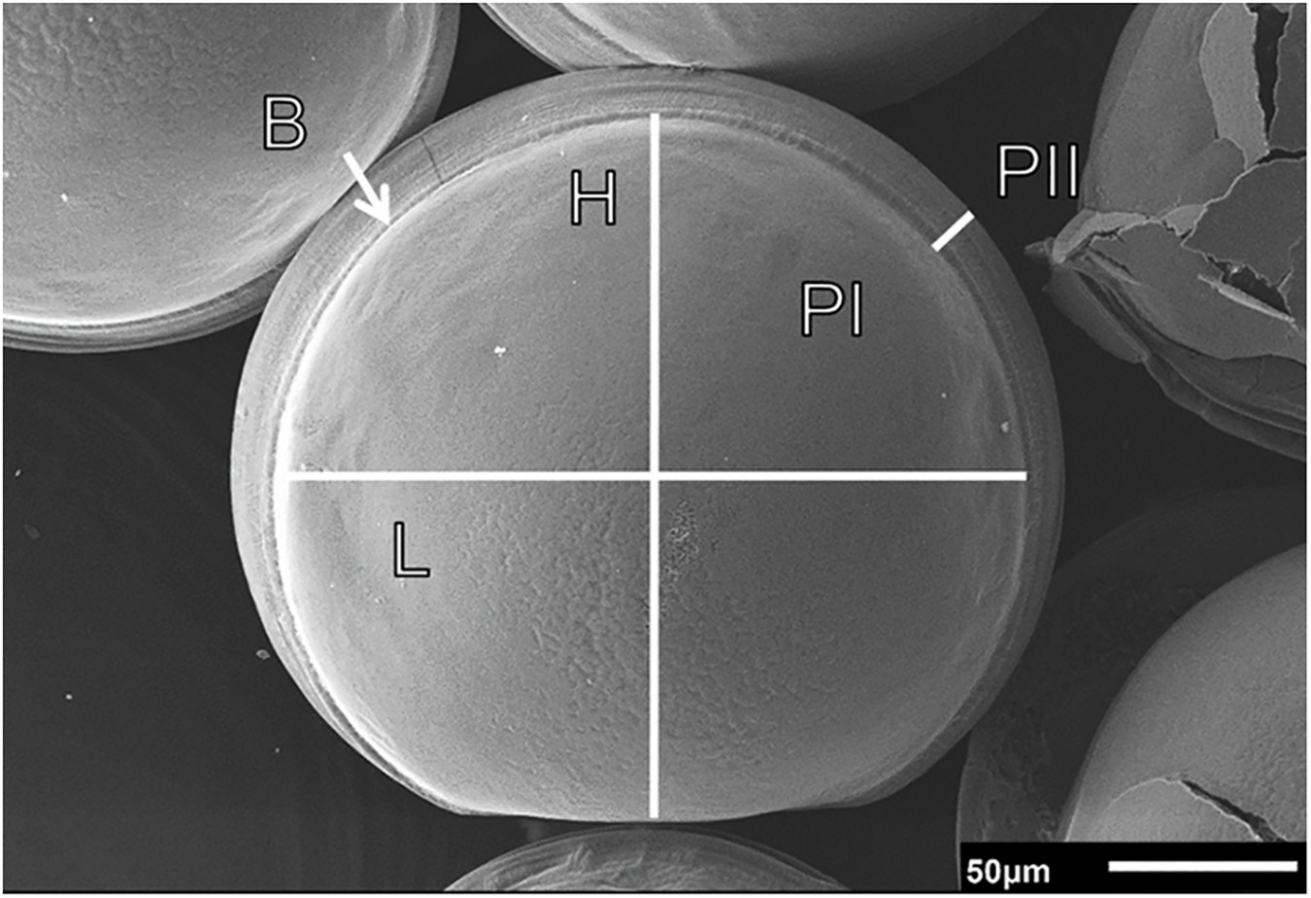
SEM image (x370) of the shell of a *L*. *elliptica* D-shape larva. Showing prodissoconch I (PI), the boundary (B) between PI and prodissoconch II (PII) and the narrow band of PII. H and L indicate measurements of shell height and length, respectively, of PI. Scale bar as indicated.

In many marine species, the first calcification that occurs during larval development utilises highly soluble amorphous calcium carbonate (ACC), which converts to aragonite over time [17], and this may be why early stages are most vulnerable to OA [18]. In bivalves, larval calcification begins at the trochophore stage when specialised ectodermal cells initiate shell formation [19]. Prodissoconch I (PI), the first larval shell, is complete when the new shell meets at the margins, completely enclosing the body and forming a straight-hinged D-shape. Larval shell deposition then continues from the shell margins in conspicuous growth bands, terminating at metamorphosis [19,20]. This part of the shell is termed Prodissoconch II (PII, Fig 1). Therefore, PI correlates to the size of the larvae at competency, while PII corresponds to shell growth and the rate of calcification between PI and metamorphosis [21].

The sizes of marine larvae, including bivalves, can be reduced in response to ocean acidification (e.g. [22,23,24]). However, reduced larval sizes often coincide with developmental delays, which suggests they are either a symptom of increased energetic costs associated with development or are due to reduced calcification (see [25,26]). Examinations of shell structures using scanning electron microscopy (SEM) have revealed abnormal development of hinge and valve structures [27], abnormal calcification [28], reduced shell integrity [25] and shell dissolution [28]. Deformities in shell hinges and valve edges may significantly reduce larval survival, while reduced shell integrity could increase susceptibility to injuries [25,27,29]. Increases in such abnormality rates are frequently observed in the SEM under ocean acidification, and it is likely that they are underestimated in studies that examine morphology using lower resolution techniques (e.g. light microscopy) [30].

In adult *Laternula elliptica*, an infaunal Antarctic bivalve with a shell comprised of aragonite, exposure of adults to increased temperatures limits metabolic and energetically demanding activities such as reburying, with prolonged exposures resulting in mortality [31,32]. Under reduced pH, adults increase oxygen consumption, protein and chitin synthase gene expression, with no impacts on mortality [33], and empty valves rapidly dissolve in acidified seawater [34]. The larvae are large and are protected by a thick egg capsule during development [35]. In a study of these larvae using light microscopy, increased temperatures resulted in faster development, and reduced occurrences of abnormalities. In the same study, reduced pH slowed development, particularly at elevated temperature, with no effect on abnormality rates [30]. However, the direct impacts of reduced pH and/or elevated temperature on shell growth and integrity in live adults or larvae are unknown.

The impacts of future climate change on shell formation and integrity in *L*. *elliptica* was studied using SEM. Shell size and quality was assessed in larvae raised under ecologically relevant scenarios of elevated temperature and reduced pH. Additionally, frequencies of abnormalities were compared to previous measurements that were determined by light microscopy.

## Materials and methods

### Collection

In November 2012, 32 adult *Laternula elliptica* were collected from the intake jetty at McMurdo Station (77°51.093’ S 166°39.931’ E), Ross Sea, Antarctica. They were transported to Wellington, New Zealand where they were held in free flowing filtered (0.1μm) seawater at -1.6°C and pH 7.98 (ambient conditions at the time of collection) until March 2013, when they were spawned to provide larvae for this experiment (and [30]). They were fed a liquid algal mix (Shellfish Diet 1800, Reed Aquaculture, USA), three times per week. Permission to conduct field sampling in Antarctica was obtained from New Zealand Ministry of Primary Industries.

### Experimental setup

Eggs were fertilised and the larvae raised under eight different temperature and pH treatments following methods detailed in [30], with three replicates per treatment. The larvae are lecithotrophic and were not fed during the course of the experiment. In addition to a temperature and pH control (-1.6°C and 7.98, respectively), elevated temperatures of -0.5 and 0.4°C were chosen to reflect end of century projections for the Ross Sea [1]. Similarly, two reduced pH treatments, 7.80 and 7.65, were chosen [1,5]. Logistical constraints allowed for a maximum of eight treatments, resulting in the temperature/pH combination 0.4°C and 7.80 not being used.

Temperature and pH manipulations were performed in eight separate header tanks, supplying insulated 4 L treatment tanks through insulated plastic tubing. pH was controlled with Omega pH controllers (Model PHCN-37-AI-230-03) using diffused food grade CO_2_. Temperature was measured using precision PT100 temperature probes, and modified via Omega CN740 controllers 2000 W connected to submersible heater elements. An automated system (see [36]) monitored temperature and pH (total hydrogen scale) in each header tank eight times per day using LabView software. This automated system measured pH spectrophotometrically and corrected aberrations from target pHs [36]. Flow was maintained at 200 ml min^-1^. On days 6, 16 and 45, water was sampled from each of the eight header tanks and preserved with HgCl_2_ for analysis of dissolved inorganic carbon (DIC) and alkalinity (A_T_) (Table 1). Saturation states of aragonite (Ω_Ar_) and calcite (Ω_Ca_) and partial pressure of CO_2_ (*p*CO_2_) at experimental temperatures and salinity were calculated from the average measured sample pH (n = 360 per treatment) and A_T_ (n = 3 per treatment) using refitted equilibrium constants [37,38] using analytical methods detailed in McGraw et al. (2010) (see Table 1).

**Table 1:** Seawater conditions for all experimental treatments.

Temp (°C)	pH	A_T_ (μmol kg^-1^)	DIC (μmol kg^-1^)	*p*CO_2_ (μatm)	Ω_Ar_	Ω_Ca_
-1.6 ± 0.01	7.97 ± 0.001	2263.3 ± 8.2	2183.8 ± 8.1	458.4 ± 1.7	1.09 ± 0.01	1.74 ± 0.01
	7.79 ± 0.001	2260.3 ± 8.9	2233.0 ± 4.0	714.4 ± 2.8	0.74 ± 0.01	1.18 ± 0.01
	7.63 ± 0.001	2266.0 ± 5.8	2271.2 ± 9.4	1045.1 ± 2.7	0.52 ± 0.01	0.84 ± 0.01
-0.5 ± 0.01	7.99 ± 0.002	2265.0 ± 8.3	2174.4 ± 3.8	460.4 ± 1.7	1.14 ± 0.01	1.82 ± 0.01
	7.80 ± 0.001	2264.8 ± 6.2	2254.0 ± 22.4	703.6 ± 1.9	0.79 ± 0.01	1.26 ± 0.01
	7.64 ± 0.001	2264.0 ± 7.2	2270.8 ± 15.0	1020.7 ± 3.3	0.56 ± 0.01	0.90 ± 0.01
0.4 ± 0.01	7.99 ± 0.001	2260.0 ± 8.4	2173.8 ± 5.8	462.2 ± 1.7	1.18 ± 0.01	1.88 ± 0.01
	7.63 ± 0.001	2260.5 ± 9.2	2260.0 ± 4.2	1052.3 ± 4.3	0.57 ± 0.01	0.91 ± 0.01

Average temperature (°C; n = 360), pH (measured on the total hydrogen scale; n = 360), total alkalinity (A_T_; n = 3) and dissolved inorganic carbon (DIC, n = 3); partial pressure of CO_2_ (*p*CO_2_), average Ω_Ar_ and Ω_Ca_ (n = 3) are calculated from A_T_ and pH. Values presented are mean ± standard error. Salinity was 34.2 psu.

### Microscopy

Larvae were raised to the D-larvae stage, the first fully shelled larva in bivalves, as described in [30]. Ninety-six hours after the point at which approximately 50% of the normally developing larvae in each treatment had reached the D-larvae stage (i.e., completed PI development); samples of approximately 200 encapsulated larvae were removed from each replicate and preserved in 90% ethanol.

Individual larvae from each treatment replicate were examined in a scanning electron microscope, (SEM: JEOL, JSM-6610LA, Japan) to determine sizes and shell morphology. Larvae were rinsed with deionised water to remove as much of the ethanol as possible. Larvae were careful excised from each egg capsule using a scalpel blade under a dissecting microscope. Isolated larvae were then placed in a drop of deionised water on carbon tape on aluminium stubs (~65 larvae per stub, with separate stubs for each treatment replicate, 24 stubs in total). The samples were air dried, and excess organic material was removed by plasma ashing (JEOL, EC-52000IC) for 30 min. Samples were stored in a vacuum desiccator until analysis, then coated with platinum (10 nm) and imaged.

Under SEM, all of the D-larvae on each stub were counted and scored for instances of severe damage or malformation. Malformation counts included larvae that deviated from the expected D-shape and also included larvae that were uncalcified. Also from each stub, 10 intact larvae with a normal D-shape were then randomly selected for analysis of shell size and quality of shell formation (magnification x350-500). The PI length (anterior to posterior) and height (hinge to ventral edge, see Fig 1) were measured for each larva to assess body size at the onset of calcification. PII length measurements are typically taken as the length across the larval body, from edge to edge. This includes the length of PI and the new growth on PII. However, as variations in PI size would influence total PII length, we measured growth of PII from the terminal edge of PI to the new shell margin. This measurement was made at three standard points along the shell edge, and these values averaged to attain a single PII length for each individual and an estimate of shell growth.

Images were imported into ImageJ (version 1.47t) and each individual was further assessed for evidence of damage and malformation of the shell surface. On each image, the total pixel areas of shell malformation, cracking or pitting were determined and expressed as a percentage of the total shell area. Values of the 10 individuals from a particular replicate were averaged to obtain one value for each parameter for each replicate prior to statistical analysis.

### Statistical analysis

All statistical analysis was performed using SPSS, version 22. Normality of the data was verified using Shapiro-Wilk’s test and equality of variance was confirmed using Levene’s test. Differences in numbers of larvae with a normal D-shape, larval body size (at PI) and shell growth (PII), as well as shell quality were related to pH and temperature by fitting the data to a general linear model using pH and temperature as fixed factors, with a pH x temperature interaction term included. If the general linear model indicated overall individual statistical significance (p < 0.05) of either temperature or pH, a post-hoc Bonferroni multiple comparison test was performed to determine effects of pH averaged over temperature and temperature averaged over pH, on larval shell sizes and quality. Where interactive effects approached significance, a one-way ANOVA was performed with treatment as a single factor, in order to determine differences between each treatment.

## Results

Reduced pH significantly increased the proportion of larvae that were malformed or were unshelled. Development and calcification of the PI and PII shell structures were observed in all treatments, despite aragonite being undersaturated at both reduced pH levels (Table 1). However, temperature and pH both had significant impacts on shell development in normal D-shaped larvae.

### Larval quality

**Fig 2:**
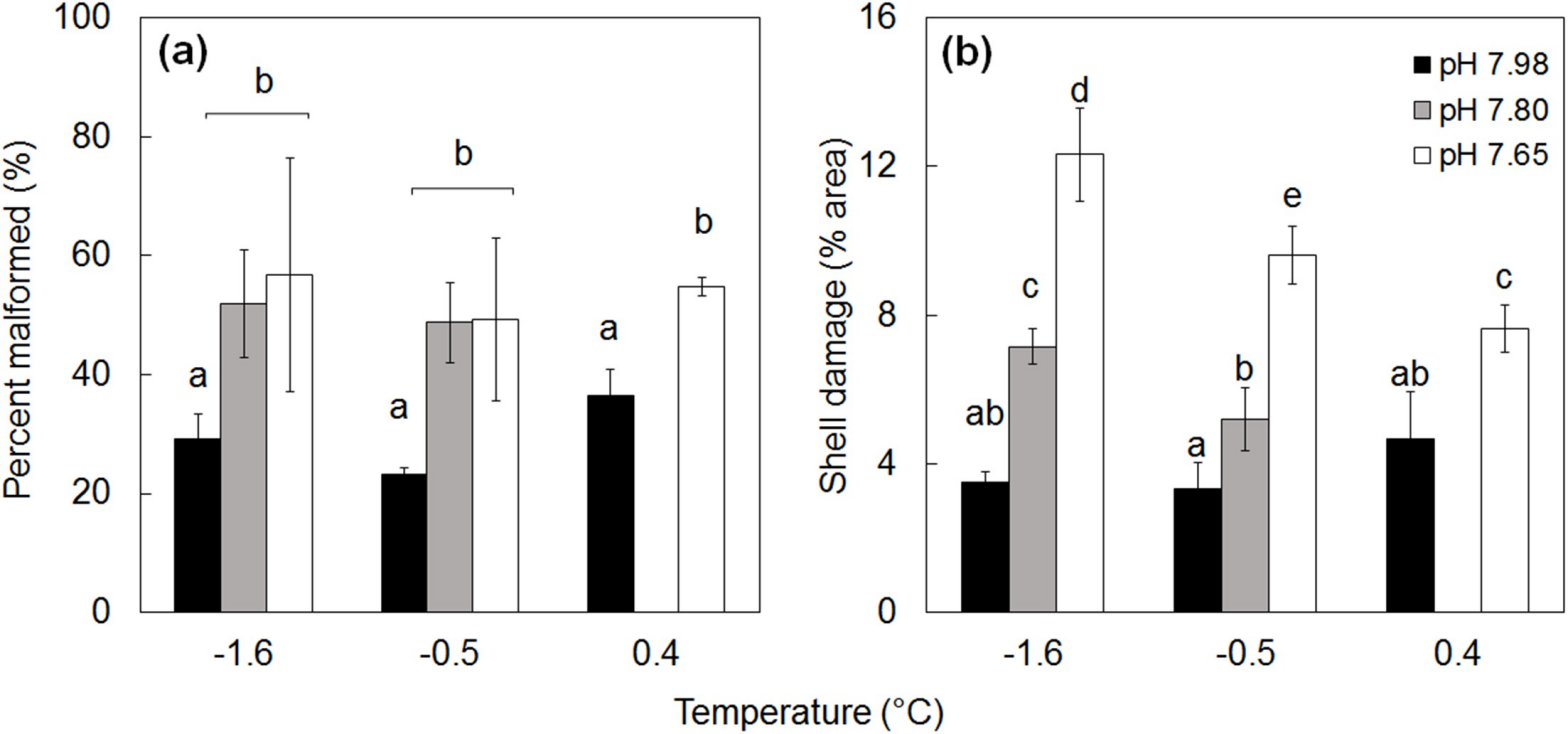
Treatment impacts on larval quality. **(**a) Percentage of malformed D-shaped larvae in each treatment and (b) shell quality assessment of normally shaped D-larvae from each treatment, where shell damage is equal to the percentage of the shell surface that is pitted, cracked or malformed. The letters above the columns indicate significance at p < 0.05. The temperature/pH combination of 0.4°C/7.80 was not used, n = 3. Error bars are standard error.

**Fig 3:**
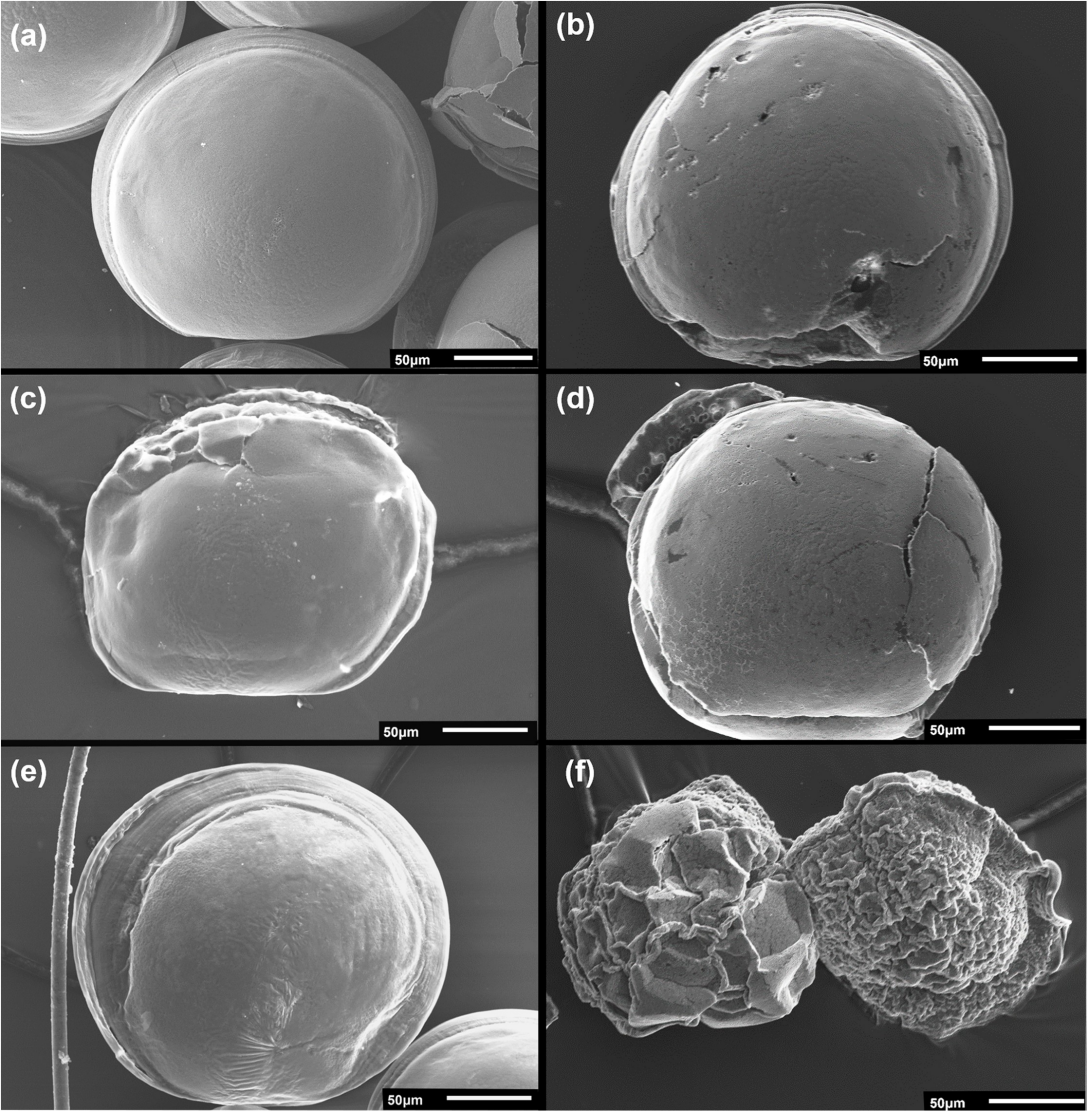
SEM images of D-larvae from various experimental treatments. (a) normally developed D-larva from the control treatment (pH 7.98 and -1.6°C, x370), and (b-e) damaged and/or malformed larvae from reduced pH (7.65) treatments at various temperatures (d: -1.6°C, x430, b: -0.5°C, x450, c and e: 0.4°C, x400), (f) extremely malformed (left) and uncalcified (right) larvae from pH 7.65, -1.6°C (x450). PI: prodissoconch I; PII: prodissoconch II. All scale bars are 50 μm.

The frequency of abnormal D-shaped larvae in each treatment, as well as shell quality, were both influenced by reduced pH (Figs 2 and 3, Table 2). Over 50% of larvae raised at pH 7.80 and 7.65 were malformed/unshelled compared to only 25–35% of those raised at ambient pH (Fig 2A). Temperature did not affect the percentage of normally developed D-shaped larvae (Table 2).

**Table 2:** Summary table of 2-way ANOVA.

	pH		Temperature		Temp x pH	
	F_2, 16_	p	F_2, 16_	p	F_3, 16_	p
Larval Quality						
Malformed/uncalcified	4.632	**0.027**	0.481	0.627	0.098	0.960
Shell Quality	157.789	**< 0.001**	14.061	0.161	23.517	0.118
Shell Measurements						
PI Height	18.966	0.617	48.191	0.310	89.711	0.238
PI Length	44.297	0.450	37.473	0.506	38.678	0.694
PII Growth	0.027	0.989	14.218	**0.016**	11.851	0.060

Summary table of 2-way ANOVA for factors pH and temperature on the proportion of larvae that were malformed/uncalcified, larval shell quality and shell measurements of D-larvae. Significant results in bold.

Damage to shell surfaces appeared as heavy pitting and cracking (Fig 3B–3D, cf. with Fig 3A, an example of a normally developing shell). Other forms of aberration included malformation of shell hinges (Fig 3B, 3D and 3E) and shell edges (Fig 3B–3E). Abnormally shaped shelled larvae were observed (Fig 3E and 3F), as were unshelled larvae (Fig 3F).

In the ten apparently normally developed D-larvae selected for closer examination, SEM revealed pitting and cracking on 3.2 to 11.6% of the total shell surface, the magnitude of which was influenced by reduced pH. Shell damage was significantly greater at pH 7.65 than at ambient pH (Table 2, Fig 2B). At the intermediate pH (7.80) shell damage was significantly different, and intermediate between, damage at ambient and 7.65 pH. Temperature had no significant impact on shell damage (Table 2).

### Shell measurements

**Fig 4:**
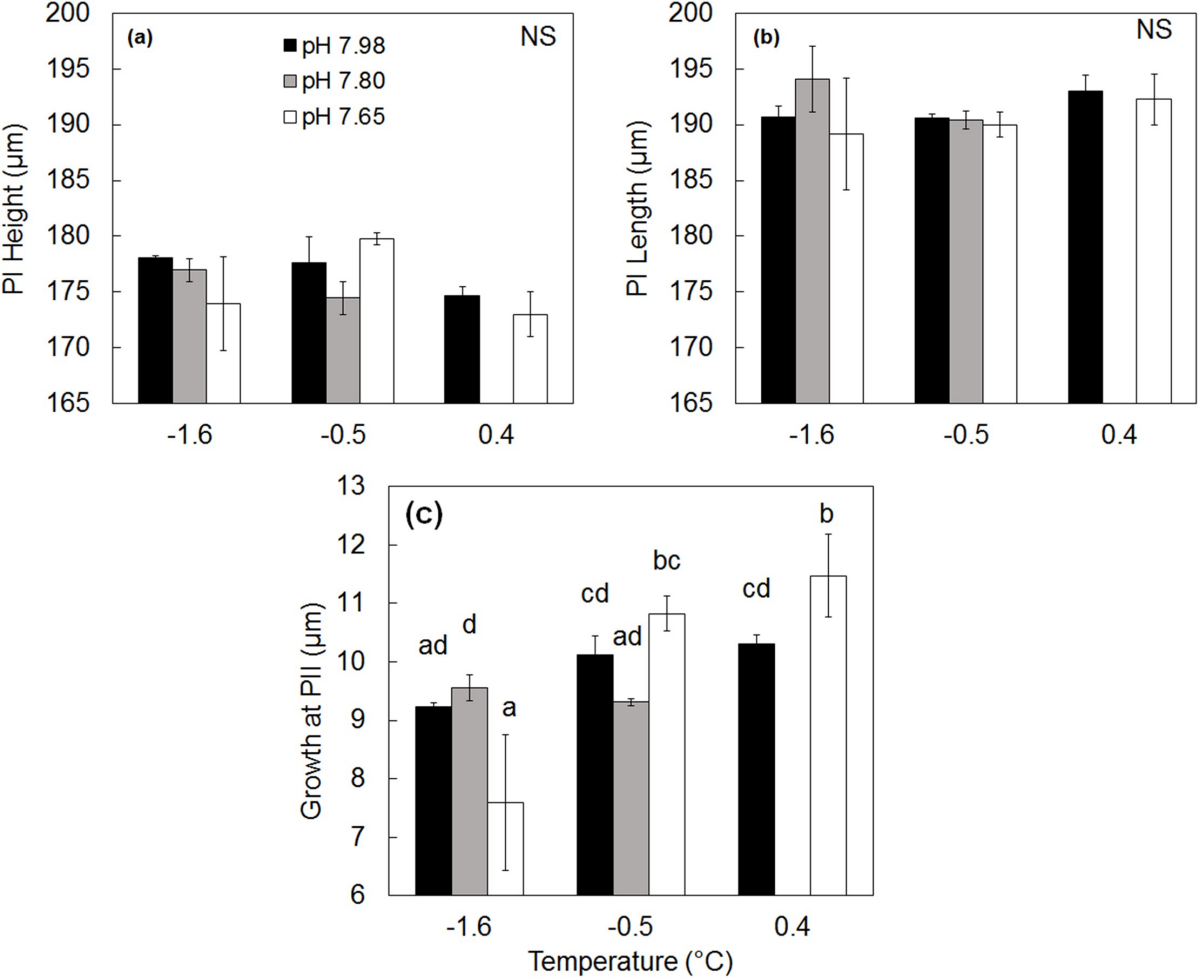
Effects of reduced pH and elevated temperatures on shell sizes. a) Shell height and b) shell length on prodissoconch I (PI) and c) on growth of prodissoconch II. Letters indicate significance as in Fig 2. The temperature/pH combination of 0.4°C/7.80 was not used. NS = no significant differences between treatments, n = 3. Error bars are standard error.

Larval shell heights at PI ranged between 173 and 180 μm, and lengths from 189 to 194 μm (Fig 4). Reduced pH and elevated temperatures had no significant impact on height or length of PI, nor therefore, on larval size at the onset of calcification (Table 2, Fig 4A and 4B).

During PII growth, larvae added 7.6–11.5 μm of new shell. This growth was significantly higher at both elevated temperatures relative to ambient, but was not significantly influenced by pH (Table 2, Fig 4C). PII growth was greatest in larvae raised at pH 7.65 and 0.4°C and lowest in those at ambient temperature and reduced pH (-1.6°C and 7.65, Fig 4C). Total shell lengths (PI+PII) ranged between 205 and 215μm, with the largest total lengths observed in larvae raised at pH 7.65 and 0.4°C.

## Discussion

Temperature and pH affected shell development and quality in *L*. *elliptica* larvae. While larval body size (measured at PI, when larvae first become fully shelled and competent) was not impacted by either stressor, shell growth (measured as PII-PI) was greater at elevated temperatures. Reduced pH negatively impacted shell quality, but did not impact growth.

In a previous study [30], abnormality rates in these larvae were assessed using light microscopy, and we concluded that abnormality rates were not impacted by pH. Larvae were apparently robust to OA, and temperature had a much greater influence on larval development in this species [30]. Here, our initial SEM evaluation of larvae with a normal D-shape indicated higher percentages of abnormal development in ambient pH cf. reduced pH at all temperatures, as well as uncalcified larvae. Furthermore, of the apparently normally developed D-shaped larvae, the higher SEM magnification showed a high percentage of those from the reduced pH treatments in fact had cracked shells and pitting (erosion) of the shell surface. Cracking and pitting was observed on all normal D-shaped larvae raised at pH 7.80 and 7.65, potentially indicating dissolution or weakening of the shell due to the undersaturation of aragonite in both reduced pH treatments. These results suggest that pH is in fact a major stressor in development for this species, with larval shells experiencing significant losses in quality, which could impair function or success.

These observations underscore the importance of methodology in determining the impacts of OA on larval development. Many investigations may inadvertently underestimate the damaging effects of pH exposure if they focus only on short term larval survival or growth, which could lead to incorrect assumptions about stress responses. SEM allows a more rigorous assessment, revealing abnormalities not immediately apparent under light microscopy (see [39]), such as dissolution and damage to fine structures (see [40,41]). SEM may also reveal changes to the crystal structure and formation indicative of changes in the calcification process (see [9]).

Abnormalities in shell morphology could significantly influence survival (see [42,43]). Reduced shell integrity could drastically impair function in larvae (see [27,29]), and subsequently reduce settlement and recruitment success. Additionally, larvae may have experienced trade-offs in muscle and tissue development in order to maintain calcification, resulting in a weaker animal [44]. Reduced muscle mass or altered shell shapes may increase the energetic cost of burying in *L*. *elliptica* juveniles. It may also expose soft tissues to damage, impacting successful settlement with negative flow-on effects to the population.

Elevated temperatures had no significant effect on shell quality, although there were indications that elevated temperature may relieve some of the negative impacts of reduced pH. For example, at pH 7.65, shell damage was reduced by 34% at the higher temperatures (Fig 2B). However, under these concurrent elevated temperature/reduced pH scenarios, shell damage was still 120% greater than in control conditions. Under projected warming and acidification, larvae will reach competency faster [30] and may experience faster shell growth, but weakness in the calcified structures could reduce recruitment and significantly increase post-metamorphic mortalities, likely overcoming any potential benefits of larger juveniles.

Shell development occurred in all reduced pH treatments despite undersaturation of aragonite, indicating calcification was not limited by saturation states. Calcification has been reported in undersaturated conditions (e.g. [45,46]), although the extent and quality of the calcium carbonate can be reduced [39,47]. Shell growth did occur in the *L*. *elliptica* larvae at reduced pH; however, the quality of the calcium carbonate structure was low. While undersaturation would promote dissolution of established calcified structures, larvae would have still been able to calcify using bicarbonate from seawater or made through the conversion of respiratory CO_2_ [48,49]. Nevertheless, calcification is still dependent on Ω at the site of calcification, and is maintained through the active pumping of ions in and out of the calcifying fluid [50]. Undersaturation may therefore increase the metabolic costs of calcification [11,51,52]. Additionally, established calcified structures would be at risk of dissolution.

Larvae in this study were sampled at different ages, but were at equivalent life history stages (96 hours post D-larvae, see Methods), and had spent similar times in PII development. The measurements of PII therefore are a reflection of the rate of calcification. Under both elevated temperatures tested, PII growth was greater than that of control temperatures, implying the larvae may have had higher rates of calcification. Larger larvae may reduce vulnerability in settled juveniles [21,53,54], indicating that recruitment in *L*. *elliptica* may be improved under future warming. The increased growth in larvae in the highest stressed treatments may due to a hormetic response in which combined stressors crossed a tolerance threshold and activated repair mechanisms (e.g. [55,56]). However, despite greater PII growth, these larvae still had significantly high damage and malformation of shell surfaces.

Under future ocean conditions, the overall impacts on *L*. *elliptica* larval shell quality and integrity due to reduced pH would likely overshadow any beneficial results of larger juveniles or faster growth at elevated temperature. Reduced survival during settlement and recruitment would limit populations of this prevalent Antarctic species.
